# Exploring Neighborhood Environments and Active Commuting in Chennai, India

**DOI:** 10.3390/ijerph15091840

**Published:** 2018-08-26

**Authors:** Deepti Adlakha, J. Aaron Hipp, James F. Sallis, Ross C. Brownson

**Affiliations:** 1 School of Natural and Built Environment, Queen’s University Belfast BT9 5AG, UK; 2 Department of Parks, Recreation, and Tourism Management, Centre for Geospatial Analytics, Centre for Human Health and the Environment, North Carolina State University, Raleigh, NC 27695-8004, USA; jahipp@ncsu.edu; 3 Department of Family Medicine and Public Health, University of California, San Diego, CA 92161, USA; jsallis@ucsd.edu; 4 Department of Family Medicine and Public Health, Australian Catholic University, Melbourne, VIC 3065, Australia; 5 Prevention Research Centre in St. Louis, Brown School, Washington University in St. Louis, St. Louis, MO 63130, USA; rbrownson@wustl.edu; 6 Department of Surgery (Division of Public Health Sciences) and Alvin J. Siteman Cancer Centre, School of Medicine, Washington University in St. Louis, St. Louis, MO 63110, USA

**Keywords:** India, active commuting, public transit, physical activity, built environment

## Abstract

Few studies assess built environment correlates of active commuting in low-and-middle-income countries (LMICs), but the different context could yield distinct findings. Policies and investments to promote active commuting remain under-developed in LMICs like India, which grapples with traffic congestion, lack of activity-supportive infrastructure, poor enforcement of traffic rules and regulations, air pollution, and overcrowding. This cross-sectional study investigated associations between home neighborhood environment characteristics and active commuting in Chennai, India. Adults (N = 370, 47.2% female, mean age = 37.9 years) were recruited from 155 wards in the metropolitan area of Chennai in southern India between January and June 2015. Participants self-reported their usual mode of commute to work, with responses recoded into three categories: (1) multi-modal or active commuting (walking and bicycling; n = 56); (2) public transit (n = 52); and (3) private transport (n = 111). Environmental attributes around participants’ homes were assessed using the Neighborhood Environment Walkability Scale for India (NEWS-India). Associations between environmental characteristics and likelihood of active commuting and public transit use were modeled using logistic regression with private transport (driving alone or carpool) as the reference category, adjusting for age, gender, and household car ownership. Consistent with other international studies, participants living in neighborhoods with a mix of land uses and a transit stop within a 10-minute walk from home were more likely to use active commuting (both *p* < 0.01). Land-use mix was significantly associated with the use of public transit compared to private transport (adjusted odds ratio (aOR) = 5.2, *p* = 0.002). Contrary to findings in high-income countries, the odds of active commuting were reduced with improved safety from crime (aOR = 0.2, *p* = 0.003), aesthetics (aOR = 0.2, *p* = 0.05), and street connectivity (aOR = 0.2, *p* = 0.003). Different environmental attributes were associated with active commuting, suggesting that these relationships are complex and may distinctly differ from those in high-income countries. Unexpected inverse associations of perceived safety from crime and aesthetics with active commuting emphasize the need for high-quality epidemiologic studies with greater context specificity in the study of physical activity in LMICs. Findings have public health implications for India and suggest that caution should be taken when translating evidence across countries.

## 1. Background

Physical inactivity is the fourth leading risk factor for global mortality and a major contributor to non-communicable diseases (NCDs) [1,2]. Worldwide, an estimated 5.3 million deaths can be attributed to insufficient physical activity [3]. This is particularly important in the context of low-and-middle-income countries (LMICs), where a bulk of the NCD burden falls—LMICs account for 80% of deaths from NCDs and physical inactivity [4]. In India, NCDs account for 53% of the disease burden, with incidence rates for heart diseases and cancer—two of the leading causes of mortality in the country—on a sharp uptick [5].

Walking and cycling are recommended forms of moderate-to-vigorous physical activity that can serve as means of travel to substitute for short car trips, and are feasible ways for people to incorporate regular physical activity into their daily lives [6]. The use of public transit usually involves walking or cycling to and from bus stops or train stations, and has the potential to contribute to overall physical activity. The promotion of walking, bicycling, use of public transport, and other non-motorized means of travel, collectively referred to as active commuting or active travel, is as a key strategy to increase physical activity [7]. Active commuting has the potential to be incorporated into people’s daily routines, and might, therefore, be more easily adopted and maintained than other forms of physical activity (especially recreational activity) [8,9,10]. Active commuters tend to achieve greater levels of physical activity than those who use automobiles [11].

Active commuting is specifically associated with reduced cardiovascular risk, lower obesity, higher physical fitness, and weight control in adults [12,13]. Despite these benefits, the mass adoption of private motorized transport and the design of cities to favor automobile use likely resulted in declining levels of active commuting and a rise in population prevalence of overweight, obesity, and related NCDs in India [14]. Our previous research from India showed that urban living was associated with lower leisure time and transport, physical activity, and increasingly sedentary lifestyles [15,16]. Active commuting may be influenced by characteristics of the neighborhood’s built environments, yet few studies quantify the associations between these factors in India.

In India, the pace of urbanization outpaced the development of basic public health services and regional infrastructure, compounding health threats from NCDs [17,18]. India’s urban areas are growing much faster than estimated previously, adding 90 million new residents in the last 10 years. By 2030, Indian cities are projected to be home to another 250 million people [19]. Along with poor health outcomes, issues of pedestrian safety, air pollution, and increasing carbon emissions are especially challenging to adapt to in urban environments already facing disparities across religious and socio-economic lines [20,21,22,23]. Successive governments prioritized investment in road infrastructure, while planning for urban growth at the local level was generally weak and haphazard. In combination, these factors resulted in urban environments with inadequate development of any public transport infrastructure and hazardous conditions for walking and bicycling in most Indian cities and towns [24].

Within India, the state of Tamil Nadu, for example, is the most urbanized state with 48.4 percent of the population living in urban areas [25], and has the highest number of diabetic cases, a majority of them being reported in the capital city of Chennai, a major commercial and industrial hub in southern India [26]. Chennai is the fourth most populous city (nine million residents) in India and the 31st most populous city in the world [27,28,29]. Unlike the three most populated Indian cities (New Delhi, Mumbai, and Kolkata) which have older and established transport infrastructures (bus rapid transit (BRT) and light rail networks), Chennai is currently undergoing changes in transport systems [30,31]. It saw a 24-fold increase in motorized vehicles since 2005, and private automobiles now constitute 55% of daily all-person trips [30,32]. The percentage of residents commuting by bus (33%), walking (26%), and bicycling (19%) in 2005 each decreased to less than 10% [30]. In addition, Chennai has the lowest walkability index (i.e., least walkable) compared to other cities in India, with pedestrians marginalized and at the bottom of the traffic food chain [33].

Land-use zoning in India is Euclidean based, which creates land-use classifications (i.e., residential, multi-family, mixed-residential, commercial, and institutional) by geographic area; however, instead of keeping those uses separate, the land uses build off of the uses in one zone to create a more integrated approach [34]. For example, in Chennai, the first land-use category called “primary residential” allows residential development, as well as cottage industries, petty shops, small farms, and schools of commerce. The “mixed-residential” zone allows everything that was allowed in primary residential, as well as other commercial establishments (e.g., banks, restaurants, cafes, and shops). The commercial land-use zone also allows primary residential and mixed residential uses [34]. While land-use zoning fosters the creation of a walkable neighbourhood with many destinations within walking distance, the connections to make that happen—the micro street grid—consisting of smaller streets and by-lanes may be missing, especially in new areas [35]. The city of Chennai has some well-used recreational bike paths, but safe bicycling facilities for transport are absent.

The World Health Organization’s Global Action Plan for the Prevention and Control of NCDs 2013–2020 stressed the need for “urban planning and transport policies to improve the accessibility, acceptability, and safety of, and supportive infrastructure for, walking and cycling,” and urged member states to ensure “the creation and preservation of built environments with a particular focus on providing infrastructure to support active commuting” [1]. In a number of high-income countries, most notably the Netherlands, Denmark, Sweden, Finland, and Germany, active commuting to work, school, or to run errands is commonplace [36]. These countries implemented effective policies and made investments in urban environments to increase active commuting. However, such efforts remain largely under-developed in LMICs like India, which suffers from fundamental concerns such as overcrowded street conditions, lack of activity-supportive infrastructure, and poor enforcement of traffic rules and regulations, all of which create barriers to active commuting [16,37].

Reversing the decline in walking and bicycling for travel-related purposes, especially for short trips, presents a major opportunity for improving physical activity worldwide. To develop effective interventions to promote active modes of transport as alternatives to car driving, an understanding of the factors associated with this particular behavior is required [7]. Public health experts contend that substantial changes in the built environment are needed if active commuting is to become a widely accepted option [7]. A few environmental and psychological factors and policies are associated with active commuting or the use of public transit, with commuting distance being the strongest and most consistent factor [38,39,40]. Additionally, the provision of sidewalks, crosswalks, and dedicated bicycle facilities on major roads, the introduction of traffic signals for pedestrians and bicyclists, and the use of traffic-calming devices is known to increase active travel [41]. However, nearly all of the evidence is from high-income countries. With the exception of some settings in South America [42,43,44] and Africa [45,46], data on active commuting in LMICs are scarce.

To our knowledge, there are no previous studies examining the associations between home neighborhood characteristics and active commuting in India. While the consequences of urban living may be exposed through a population’s health, the underlying causes or amplifications of health problems are often rooted in conditions best addressed through non-public health pathways such as neighborhood design and planning, as explored in this study. The objective of the current study was to examine built environment correlates of usual commuting modes in an adult population in Chennai, India.

## 2. Methods

### 2.1. Sampling and Recruitment

Participants (N = 370, female = 47.2%) were recruited from 155 wards in the metropolitan area of Chennai in southern India (5th largest city; population as per 2011 Census = 8,653,521) between December 2014 and June 2015. Wards were stratified to maximize variance in neighborhood walkability and socio-economic status (SES) to enhance the representativeness of the sample because low-SES populations tend to be underrepresented in studies of this nature [47,48]. 

Inclusion and exclusion criteria for participants were based on studies conducted by by the International Physical Activity and Environment Network (IPEN) study protocol [49] in LMICs such as Africa, Brazil, and China [46,49,50]. Eligibility criteria included the following: (i) current residents of the Chennai metropolitan area; (ii) residents for at least six months; (iii) 18–65 years of age; (iv) being able and willing to answer questions in English or Tamil (official language in the study region); (v) not having any disability that prevented independent walking; and (vi) no visible signs of cognitive impairment. One individual per household was recruited to ensure independence of observations. The sample size was determined using a moderate-to-large effect size (effect size statistic (d) = 0.75), which is greater than what was used in previous IPEN studies in LMIC contexts [51,52]. To reduce the risk of bias, this study adopted a sampling strategy to represent diverse environments. In addition, the use of reliable measures and standardized protocols for data collection, including training of study personnel and minimized inter-observer variability when multiple field workers were gathering and entering data. Details of neighborhood stratification, sampling, recruitment, and survey properties were described in detail elsewhere [53].

### 2.2. Ethics Approval and Consent to Participate

All study procedures were approved by the Institutional Review Board, Human Research Protection Office, Washington University in St. Louis, USA (study ID: 201410052). This study presented no more than minimal risk to participants, and was given an exempt status by the Human Research Protection Office at the WUSTL. It involved no procedures for which written consent is normally required outside of the research context. No names or identifying information about participants were collected in the survey. All participants were provided with a written statement regarding the research study, in the form of an information sheet. The WUSTL IRB approved this study without a requirement for written consent.

## 3. Measures

### 3.1. Main Outcome

The main outcome of interest was the commuting mode. Participants self-reported their usual mode of commuting to work. The dependent variable response options included the following: walk, bicycle, public transport (bus, train, or auto rickshaw), private transport (car or motorcycle), and a combination of walk/bicycle with public and private transport modes. Participants could select multiple modes. To account for health benefits, unless a walking trip was at least 10 min (e.g., to or from a public transit stop or parking lot), participants were asked not to report it as a “walk” [6]. Commuting mode was recoded into three categories: (1) multi-modal or active commuting (walking and bicycling); (2) public transit; and (3) private transport. Participants who reported multi-modal travel (using both active and non-active modes) were grouped together with active commuting to capture the active components of multi-modal commuters. Participants who reported using multi-modal travel involving only non-active modes were grouped together with private transport.

### 3.2. Independent Variables and Covariates

#### 3.2.1. Socio-Demographic and Individual Characteristics

Self-reported data on age, gender, race, marital status, education, household income, number of vehicles in the household, number of children younger than 18 years old in the household, and chronic conditions including heart disease, diabetes, and cancer were elicited from participants. The sociodemographic characteristics were categorized into 2–4 categories where appropriate (Table 1).

#### 3.2.2. Home Neighborhood Built Environment Features

Built environment features of participants’ home neighborhoods were assessed using the Neighborhood Environment Walkability Scale for India (NEWS-India) consisting of 91 items grouped into eight subscales as listed in Table 2 [53]. NEWS item scoring and subscale score calculations followed the NEWS-Adult scoring scheme recommended by the IPEN study protocol [49]. Four-point Likert-type scale response options for all NEWS-India items ranging from 1 (strongly agree) to 4 (strongly disagree) were combined as “agree” (strongly agree, agree) and “disagree” (disagree, strongly disagree). All NEWS-India items were positively scored to ensure that a higher score denoted a more activity-supportive neighborhood. Based on scoring procedures and results from confirmatory factor analyses of NEWS conducted previously [36,49], two aggregate scores (mean of subscales) were computed: (i) a conceptual NEWS-India score including variables that are known to be related to active commuting (Table 1, subscales a–e, excluding aesthetics, safety from crime, and safety from traffic since they were more likely to affect leisure physical activity); and (ii) a composite score specific to Chennai consisting of only the built environment variables that were significantly positive or not significant (Table 1, subscales a, b, and e). Test–retest reliability of NEWS-India items were previously established, with reliability coefficients that were generally high (intraclass correlation coefficient (ICC) = 0.48 to 0.99) with almost perfect strength of agreement, indicating that the items were generally reliable [53].

## 4. Data Analysis

Data were analyzed in two multiple logistic regression models with private transport (i.e., driving alone or carpool) as the reference commuting mode to examine the following: (1) the correlates associated with using multi-modal or active commuting (Table 3), and (2) the correlates associated with using public transit (Table 4). A summary of the variables used in the multiple logistic regression models is presented in Table 1. Descriptive statistics of the sample population are presented in Table 2. Unadjusted and adjusted odds ratios (aORs) are presented in Table 3 and Table 4. All models were adjusted for age, gender, and household car ownership. These covariates were selected to control for the confounding effects of these variables as shown in similar studies conducted in LMICs [36,49,55]. Data were analyzed using the Statistical Package for the Social Sciences (SPSS) version 21 (IBM, Armonk, NY, USA). By default, missing values were removed in SPSS in a list-wise manner, leaving only cases with all variables in the final regression model. 

## 5. Results

The availability of a transit stop within a 10-minute walk from homes was strongly associated with an increase in the likelihood of active commuting (aOR = 5.0, *p* = 0.003) and a trend toward a higher likelihood of public transit usage, although this relationship was not significant (aOR = 2.5, *p* = 0.08). Among the built environment characteristics, a mix of land uses was related to higher active commuting (aOR = 6.8, *p* = 0.001) and to the use of public transit compared to private transport (aOR = 5.2, *p* = 0.002). The availability of supportive infrastructure for walking and cycling tended to improve the odds of active commuting (aOR = 2.6, *p* = 0.07), but this relationship was not significant. Aesthetics (aOR = 0.2, *p* = 0.003) and safety from crime (aOR = 0.2, *p* = 0.05) were significantly associated with a reduced likelihood of active commuting. Street connectivity also was associated with a reduced likelihood of active commuting (aOR = 0.2, *p* = 0.003) and public transit use (aOR = 0.2, *p* = 0.004). Shorter commuting distances (1–5 km) explained the uptake of active commuting (aOR = 6.4, *p* = 0.09), but this relationship was not significant in the adjusted model. Commuting distance was not associated with use of public transit.

In unadjusted models, the NEWS-India conceptual score and the NEWS-Chennai composite score significantly predicted an increase in odds of active commuting by approximately two times (OR = 2.1, *p* = 0.03), but they were not associated with the use of public transit (OR = 1.3, *p* = 0.5). In adjusted models, both scores were neither associated with active commuting (aOR = 1.1, *p* = 0.9) nor public transit use (aOR = 0.8, *p* = 0.7).

## 6. Discussion

### 6.1. Principal Findings

This exploratory study provides new evidence on neighborhood environment attributes associated with active commuting in India, a rapidly urbanizing LMIC context. A large majority of the prior evidence on built environment correlates of active commuting was limited to high-income countries, and more recently, some LMICs in South America and Africa [56,57]. Findings from this preliminary study in India highlight some contrasting results in comparison with those reported in high-income countries, emphasizing the need for more high-quality epidemiologic studies from LMICs. In general, we found that supportive environments were associated with more active commuting and public transit use. Residents living in neighborhoods with a diversity of destinations and the availability of walking and bicycling facilities were more likely to choose active modes of commuting. Access to public transport in the neighborhood can act as a facilitator for a more active lifestyle among its residents. However, some surprising results were that street connectivity, safety from crime, and aesthetics were inversely related to active commuting. These results raise the possibility that built environment attributes found to facilitate active commuting in higher-income countries do not fully generalize to LMICs. Given that active commuting is decreasing in LMICs while NCDs are increasing, there is a need for more research in LMICs to inform context-specific built environment strategies to increase physical activity.

### 6.2. Comparison with Existing Literature

Several land-use and transportation factors are known to be associated with active commuting [9,56,58,59]. Consistent with other international studies from both high-income countries and LMICs, the strongest correlate of active commuting and use of public transit was land-use mix [43,60,61,62]. Results from the present study confirmed previously reported associations that the availability of a mix of destinations around homes—shops and stores, and recreation facilities such as parks, walking trails, bike paths, and recreational centers—was associated with a higher likelihood of active commuting or multi-modal commuting. Lower commuting distances and the availability of walking and cycling infrastructure were also associated with active commuting, which aligns with previous research. A recent study on longitudinal associations on built environment characteristics found that supportive environments predicted an uptake of active commuting—participants living in neighborhoods with a greater density of employment locations were more likely to engage in and maintain their active commuting [10]. In the United States (U.S.), two studies of university students indicated that the farther the distance or the longer the commuting times between home and university, the lower the likelihood was of choosing to use an active commuting mode [63,64].

In contrast with findings from high-income countries, improved safety from crime and aesthetics reduced the odds of active commuting. Previous research yielded similar inconclusive results acknowledging that the impact of perceived safety from crime and aesthetics on physical activity behaviors in residential neighborhoods needs careful examination [65]. Some studies suggest that higher fear of crime was associated with lower levels of walking, and higher perceived danger for pedestrians and cyclists was associated with increases in car use [66], although some studies found no associations between changes in perceptions of safety and walking [67]. These mixed findings may relate to the complexity of measuring crime, which is likely to be sensitive to time of occurrence, location, people’s perceptions, and social context [68].

In our analyses, short commuting distance from home to work demonstrated a strong association with active commuting in unadjusted models, which confirms previous research [58], but it did not remain significant in adjusted models. Studies showed distance from home to work was a strong predictor of uptake and maintenance of active commuting, and local planners may be able to co-locate new residential developments and workplaces, thus reducing the distances required to travel to work [10]. In unadjusted models with NEWS-India aggregate scores, the active commuting model was significant, while the public transit model was not. An explanation for this may be that the built environment variables are more relevant to walking and bicycling rather than motorized transport [69]. It was surprising that the Chennai-specific model did not perform better than the full conceptual model that included some variables with inverse associations. The weak performance of the conceptual and composite variable could be due to the inclusion of several variables with inverse associations with active commuting (e.g., street connectivity, land-use mix—access), suggesting that a different combination of environmental variables may be optimal in some LMIC contexts. Furthermore, the loss of significance in adjusted models could be due to the inclusion of car ownership that may be acting as a mediating variable between the built environment and commute mode choices. After car ownership, age and gender may be the next most influential factors as studies found that women exercise less compared to men and that people are less active as they get older [70,71]. In studies from LMICs like Mexico, Colombia, and Nigeria, car ownership was negatively associated with transport-based activity levels—higher levels of transport-based activity levels tended to occur in the non-vehicle owners, and may be strongly driven by necessity [57,59,72]. Thus, evidence is growing to support an interpretation that car owners in LMICs will drive regardless of whether or not the neighborhood environment supports active transport. Researchers recommended that this inverse relationship calls for a need-based framework for understanding active commuting in LMICs, versus the more common choice-based framework [50]. More research is needed to understand and assess additional factors (e.g., socio-economic status, car ownership, commuting patterns, and travel distances) and their relationship with active commuting across larger geographical areas and over extended periods of time in LMIC contexts.

## 7. Limitations

The cross-sectional study design and a relatively small sample from a single city in India preclude causal inference and generalizability of results [73]. Given the exploratory nature of this article, findings from Chennai in India may not generalizable to other cities in India. Demographic differences between the neighborhoods sampled, residual confounding, and self-selection of individuals into walkable neighborhoods may further limit generalizability [74,75,76,77].

Self-reported measures are subject to bias (e.g., overestimation, social desirability of physical activity, and physically active people noticing more built environment features and commuting destinations) [78]. Recent studies used accelerometers and global positioning system (GPS) devices to objectively measure physical activity [59,79]. However, given the early stages of this research in India, the present study provides initial evidence of active commuting and built environment attributes. A lack of consensus on measuring domain-specific activity (e.g., inadequate details on types of physical activity and their components, and a lack of reliable measurement tools) is another limitation of this study and physical activity literature in LMICs [80,81]. Despite these limitations and a relatively small sample, this study has notable strengths. To the best of our knowledge, this study is among the first to use the validated NEWS-India tool to document built environment features and active commuting behaviors in India.

## 8. Implications

The role of supportive infrastructure in neighborhoods (e.g., sidewalks, bicycle lanes, and transit stops) for the incorporation of transport-related physical activity into a daily routine is a key take-home message of this study. Even though some findings were different from those in higher-income countries, mixed land use, pedestrian and bicycling infrastructure, and proximity to transit were all positively related to active transport in Chennai, India. These findings may suggest that improved urban design and transport options could improve active transport and physical activity in an Indian context. Within India, this study complements the objectives of on-going national government schemes in India, such as the Smart Cities Mission (http://smartcities.gov.in/content/), the Swachh Bharat Mission (http://www.swachhbharaturban.in/sbm/home/), and the Atal Mission for Rejuvenation and Urban Transformation (AMRUT, https://amrut.gov.in/), in aspects of urban land use and health promotion. Present results simultaneously inform interlinked agendas, including spatial growth management, effective land use, formulation of transport policies to ease traffic congestion, vision zero initiatives, and clean air policies, with the potential to establish and extend the field of active living research in India [82].

## 9. Conclusions

A significant portion of the NCD burden is concentrated in LMICs; however, research on environmental correlates of active commuting is lacking that represents the diverse contexts of LMICs. The costs to the wider Indian economy from a heavy reliance on the private car as a mode of transportation run into tens of billions of dollars every year [23,83]. The evidence base for increasing the uptake of active commuting is extensive [84], and active commuting presents an untapped opportunity to achieve regular physical activity. Achieving increased active commuting requires coordinated work among various stakeholders, but the benefits are numerous, and go beyond physical inactivity and sedentary lifestyles, including positive impacts on traffic congestion, air pollution, and carbon emissions [84,85]. However, the present study adds to other evidence from LMICs [35,37,48] that car ownership appears to negate any effect of walkable neighborhood design on active commuting. Thus, special efforts may be needed to encourage car owners in LMICs to walk for transport, in addition to activity-supportive built environments.

The science of measuring and improving the built environment related to physical activity is inherently multidisciplinary. It is important to recognize that land-use mix is not just a research instrument for examining the impact of environments on physical activity, but is a valid planning tool that practitioners and policy makers can use to make neighborhoods more conducive to active lifestyles [60]. Accurate built environment measures and development of more precise benchmarks through further research may be needed to assist informed decisions about the planning and design of activity-friendly neighborhoods in India, which may be helpful in improving the long-term health of residents. In India, much remains to be done to measure, adapt, and design built environments to promote active commuting. It is, therefore, essential to promote more collaboration among public health investigators, as well as those from non-public health disciplines (e.g., urban design/planning, transportation engineering, and sociology) to identify overlapping research priorities. There is a clear need for public health professionals to work closely with other disciplines on research, policy, and practice that will lead to joint efforts to meet societal needs.

Future progress depends on forging effective collaborations across disciplines, improving training and education, increasing the resources provided by funding agencies, and capacity-building efforts that cross traditional disciplinary boundaries to facilitate knowledge exchange between developed countries and LMICs. Local evidence can guide regional or national policies to improve built environment factors that directly or indirectly affect transport mode choices, having the potential to increase physically active transport. An integrated effort toward encouraging walking, cycling, and public transport use, while reducing dependency on cars, is a promising strategy to significantly increase active commuting, and therefore, physical activity in LMICs.

## Figures and Tables

**Table 1:** Descriptive statistics of the sample population (n = 367).

Descriptive Characteristics	Statistic
Age (in years), mean (SD)	37.9 (15.3)
Gender, n (%)	
Female	199 (54.2)
Male	166 (45.2)
Marital Status, n (%)	
Married	226 (61.2)
Not married	143 (38.8)
Religion, n (%)	
Hindu	304 (82.2)
non-Hindu	65 (17.6)
Educational Level, n (%)	
Uneducated	48 (13.0)
Primary–middle school	57 (15.5)
High school or diploma	79 (21.5)
Graduate or professional	184 (49.7)
Monthly Family Income in United States (U.S.) Dollars, n (%)	
≤80	74 (25.3)
81–200	43 (14.7)
201–549	24 (8.2)
≥550	152 (51.9)
Work Status, n (%)	
Unemployed	134 (37.5)
Blue collar	112 (31.4)
White collar	111 (31.1)
Physical Activity Levels, n (%)	
Walk	40 (10.8)
Bicycle	7 (1.9)
Public transport (Bus, local/suburban train, or auto rickshaw)	52 (14.1)
Private transport (car, motorcycle, or scooter)	103 (27.8)
Multi-modal	21 (5.7)

Note: 1 US Dollar = approximately 65.69 Indian Rupees (average currency exchange rate, January–April 2015); cut-off values in table based on socio-economic status (SES) classification for India by Gururaj and Maheshwaran (2014) [54].

**Table 2:** Summary of variables used in multiple logistic regression models.

Type of Variable	Model 1	Model 2
Dependent Variables	Active travel vs. private transport (reference category)	Public transit vs. private transport (reference category)
Independent Variables	Public transit access (transit stop within a 10-min walk from home) Commute distance to work (1–5 km, 5.1–10 km, 10.1–15 km, or >15 km) Neighborhood Environment Walkability Scale for India (NEWS-India) subscale scores (mean of items): Residential density (7 items) Land-use mix–diversity (43 items) Land-use mix–access (7 items) Street connectivity (5 items) Infrastructure for walking and cycling (13 items) Safety from traffic (6 items) Safety from crime (4 items) Aesthetics (6 items) NEWS-India aggregate score (mean of subscales a–e) ^1^	
Covariate/Controls	Age (continuous) Gender (dichotomous) Household car/motor vehicle ownership (dichotomous)	

^1^ NEWS-India aggregate score excluding aesthetics, safety from crime, and safety from traffic.

**Table 3:** Crude and adjusted odds ratios examining associations between multi-modal or active commuting vs. private transport and home neighborhood supports in Chennai, India (Model 1).

Reference: Private Transport (n = 111)	Multi-Modal or Active Commuting (n = 56)					
	Unadjusted logistic regressions			Adjusted logistic regressions		
	OR	95% CIs	*p*	aOR	95% CIs	*p*
Public transit access						
Transit stop within a 10-min walk from home (reference: Disagree)						
Agree	4.5	2.1–9.6	<0.001	5.0	1.7–14.4	0.003
Commuting distance (reference: >15 km)						
1–5 km	22.5	2.8–179.4	0.003	6.4	0.7–55.4	0.09
5.1–10 km	2.1	0.2–25.3	0.6	0.0	0.0–0.0	1.0
10.1–15 km	1.9	0.2–22.7	0.6	1.6	0.1–20.4	0.7
Residential density (reference: Low)						
High	2.2	1.1–4.2	0.02	1.1	0.4–2.7	0.9
Land-use mix—diversity (reference: Low)						
High	7.7	3.5–17.0	<0.001	6.8	2.3–20.6	0.001
Land-use mix—access (reference: Disagree)						
Agree	0.5	0.2–1.0	0.05	0.4	0.1–1.2	0.1
Street connectivity (reference: Disagree)						
Agree	0.2	0.1–0.4	<0.001	0.2	0.1–0.6	0.003
Infrastructure for walking or bicycling (reference: Disagree)						
Agree	2.1	1.0–4.5	0.05	2.6	0.9–7.1	0.07
Safety from traffic (reference: Disagree)						
Agree	0.8	0.4–1.7	0.6	0.8	0.3–2.3	0.7
Safety from crime (reference: Disagree)						
Agree	0.2	0.1–0.4	<0.001	0.2	0.1–0.6	0.003
Aesthetics						
Agree	0.3	0.1–0.7	0.01	0.2	0.0–1.0	0.05
NEWS-India conceptual score (reference: Low)	2.1	1.1–4.1	0.03	1.1	0.4–2.6	0.9
High						
NEWS-Chennai composite score (reference: Low)						
High	2.1	1.1–4.1	0.03	1.1	0.4–2.6	0.9

Note: aOR—adjusted odds ratios; adjusted for age (continuous), gender (categorical), and household car ownership (categorical). CIs—confidence intervals.

**Table 4:** Crude and adjusted odds ratios examining the associations between public vs. private transport and home neighborhood supports in Chennai, India (Model 2).

Reference: Private Transport (n = 111)	Public Transit (n = 52)					
	Unadjusted logistic regressions			Adjusted logistic regressions		
	OR	95% CIs	*p*	aOR	95% CIs	*p*
Public transit access						
Transit stop within a 10-min walk from home (reference: Disagree)						
Agree	3.7	1.7–8.1	0.001	2.5	0.9–6.9	0.08
Commuting distance (reference: >15 km)						
1–5 km	0.8	0.3–2.0	0.6	0.3	0.1–1.2	0.1
5.1–10 km	0.8	0.3–2.3	0.7	0.4	0.1–1.5	0.2
10.1–15 km	0.6	0.2–1.9	0.4	0.3	0.1–1.3	0.1
Residential density (reference: Low)						
High	1.2	0.6–2.4	0.6	0.8	0.3–1.8	0.5
Land-use mix—diversity (reference: Low)						
High	5.2	2.3–11.7	<0.001	5.2	1.8–14.9	0.002
Land-use mix—access (reference: Disagree)						
Agree	0.4	0.2–0.9	0.02	0.6	0.2–1.7	0.4
Street connectivity (reference: Disagree)						
Agree	0.2	0.1–0.4	<0.001	0.2	0.1–0.6	0.004
Infrastructure for walking or bicycling (reference: Disagree)						
Agree	0.9	0.4–2.2	0.8	1.3	0.4–3.7	0.7
Safety from traffic (reference: Disagree)						
Agree	1.7	0.9–3.4	0.1	1.9	0.8–4.5	0.2
Safety from crime (reference: Disagree)						
Agree	0.6	0.3–1.1	0.09	0.8	0.4–2.0	0.7
Aesthetics						
Agree	0.7	0.3–1.6	0.4	1.0	0.4–2.6	0.9
NEWS-India conceptual score (reference: Low)	1.3	0.7–2.4	0.5	0.8	0.4–2.0	0.7
High						
NEWS-Chennai composite score (reference: Low)						
High	1.3	0.7–2.4	0.5	0.8	0.4–2.0	0.7

Note: aOR—adjusted odds ratios; adjusted for age (continuous), gender (categorical), and household car ownership (categorical). CIs—confidence intervals.

## Availability of Data and Material

The datasets used and/or analyzed during the current study are available from the corresponding author upon reasonable request.

## Abbreviations

NCDs	non-communicable diseases
LMICs	low-and-middle-income countries
